# Automated image analysis for quantification of filamentous bacteria

**DOI:** 10.1186/s12866-015-0583-5

**Published:** 2015-11-04

**Authors:** Marlene Fredborg, Flemming S. Rosenvinge, Erik Spillum, Stine Kroghsbo, Mikala Wang, Teis E. Sondergaard

**Affiliations:** Department of Animal Science, Faculty of Science and Technology, Aarhus University, Blichers Allé 20, 8830 Tjele, Denmark; Department of Clinical Microbiology, Vejle Hospital, Kabbeltoft 25, 7100 Vejle, Denmark; Philips BioCell, Gydevang 42, 3450 Allerød, Denmark; Department of Clinical Microbiology, Aarhus University Hospital, Palle Juul-Jensens Boulevard 99, 8200 Aarhus, Denmark; Department of Biotechnology, Chemistry and Environmental Engineering, Aalborg University, Sohngaardsholmsvej 49, 9000 Aalborg, Denmark

**Keywords:** Morphology, Antibiotic-induced filamentation, Digital microscopy, Time-lapse imaging, Cell elongation, *Escherichia coli*

## Abstract

**Background:**

Antibiotics of the β-lactam group are able to alter the shape of the bacterial cell wall, e.g. filamentation or a spheroplast formation. Early determination of antimicrobial susceptibility may be complicated by filamentation of bacteria as this can be falsely interpreted as growth in systems relying on colorimetry or turbidometry (such as Vitek-2, Phoenix, MicroScan WalkAway). The objective was to examine an automated image analysis algorithm for quantification of filamentous bacteria using the 3D digital microscopy imaging system, oCelloScope.

**Results:**

Three *E. coli* strains displaying different resistant profiles and differences in filamentation kinetics were used to study a novel image analysis algorithm to quantify length of bacteria and bacterial filamentation. A total of 12 β-lactam antibiotics or β-lactam–β-lactamase inhibitor combinations were analyzed for their ability to induce filamentation. Filamentation peaked at approximately 120 min with an average cell length of 30 μm.

**Conclusion:**

The automated image analysis algorithm showed a clear ability to rapidly detect and quantify β-lactam-induced filamentation in *E. coli*. This rapid determination of β-lactam-mediated morphological alterations may facilitate future development of fast and accurate AST systems, which in turn will enable early targeted antimicrobial therapy. Therefore, rapid detection of β-lactam-mediated morphological changes may have important clinical implications.

**Electronic supplementary material:**

The online version of this article (doi:10.1186/s12866-015-0583-5) contains supplementary material, which is available to authorized users.

## Background

**Fig. 1:**
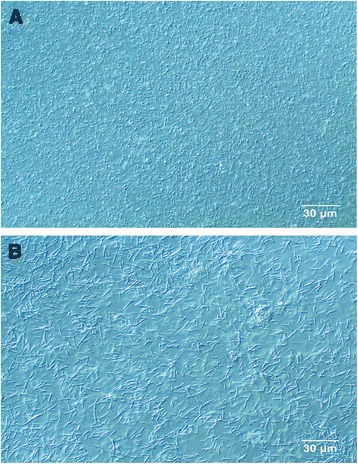
Antibiotic-induced filamentation in *E. coli* by light microscopy. Antibiotic-induced filamentation of *E. coli* ATCC259222 treated with cefotaxime (8 mg/L) visualized in light microscopy with a 10x (**a**) and a 100x (**b**) magnification

Antibiotics of the β-lactam group are able to alter the shape of the bacterial cell wall, e.g. inducing filamentation or a spherical shape of rod-shaped bacteria (Fig. 1) [1–3]. Filamentation occurs when cell division is blocked while growth continues leading to the formation of filamentous bacteria with lengths of up to 50 times their bacillary counterparts [4]. Filamentous morphology provides survival advantages: inhibits phagocytic uptake by immune cells [5], promote survival within host tissue [6, 7], and decrease susceptibility to certain antimicrobial agents [4]. However, the transformation to grow in a filamentous manner may not be enough to overcome a certain environmental stress. In these cases, elongation of the cell shape is followed by the presence of holes in the cell wall, indicating irreversible damage, subsequently resulting in exit of cytoplasmic material producing ghost cells which will finally lyse and disappear [8]. Gram negative bacteria exposed to β-lactam antibiotics frequently display a change towards a filamentatious morphology which may allow for survival until the antibiotic is diluted or becomes inactive [6]. Even though filamentation cannot be directly correlated to antibiotic susceptibility or resistance, it is pivotal knowledge in relation to early antimicrobial susceptibility testing (AST).

Beta-lactam antimicrobials are widely used to treat infections; however, the alarming spread of β-lactam resistance is increasingly reported worldwide and represents a major health issue [9]. Among the most important resistance mechanisms to penicillins and cephalosporins in *Enterobacteriaceae* are extended spectrum β-lactamases (ESBL) [10]. As infections caused by ESBL-producing pathogens may not be responsive to treatment with most penicillins and cephalosporins, and as the genes encoding this resistance mechanism are harbored on plasmids and readily transferrable, their presence in a hospital setting should be identified quickly to ensure appropriate antimicrobial therapy [11, 12].

In a previous study, we demonstrated the oCelloScope system as a fast and sensitive AST method [13]. The oCelloScope system provides information on growth kinetics based on advanced algorithms analyzing image material. In this study, we present an image analysis algorithm quantifying the length of bacteria and thus changes in bacterial morphology. This is particularly important when testing antibiotics such as penicillins, cephalosporins, carbapenems, and monobactam. β-lactam-antibiotics are known to induce morphological changes in bacteria (e.g. filamentation or spheroplast formation) [8]. Rapid detection of these changes may therefore have important clinical implications.

## Methods

### Organisms

To examine the antibiotic-induced morphological changes, three *E. coli* strains were used displaying differences in resistance profiles and filamentation kinetics: *E. coli* reference strain ATCC25922 (no resistance), a multi-drug resistant ESBL-producing clinical isolate (aztreonam-R, ceftazidime-R, piperacillin-R and ticarcillin-R), and an *E. coli* from a positive blood culture (no resistance). Ethical approval was not required for this study according to Danish Law.

### Growth conditions

The *E. coli* reference strain and the ESBL *E. coli* were grown under atmospheric conditions at 37 °C in cation adjusted Mueller-Hinton broth with TES (CAMHBT) overnight. The blood culture *E. coli* strain was isolated from a blood sample from a patient with bacteremia which were collected in standard aerobe non-charcoal BacT/ALERT FA blood culture bottles (bioMérieux, Marcy l’Etoile, France), and handled according to the manufacturer’s instructions. Blood culture bottles were incubated in the BacT/ALERT 3D system (bioMérieux, Marcy-l’Etoile, France) at Aarhus University hospital (Aarhus, Denmark). The blood culture sample was prepared by centrifugation at 200 × *g* for 5 min (Sigma 3–18 k, 12171 rotor, Buch & Holm, Herlev, Denmark) to remove human blood cells, followed by resuspension of the bacterial pellet in CAMHBT.

### Antibiotic-induced morphological changes

Bacterial suspensions were diluted to McFarland 0.5 based on OD_600_ measurements using a UV-3100 PC Spectrophotometer (VWR, Herlev, Denmark) and subsequently diluted in cation adjusted Mueller-Hinton broth to a final bacterial cell suspension of approximately 1 × 10^5^ bacteria/mL. Sensititre® NF plates (Thermo Scientific, Roskilde, Denmark) were used to examine antibiotic-induced morphological changes. The Sensititre NF plates contain a total of 23 antibiotics of which 12 are β-lactam antibiotics or β-lactam–β-lactamase inhibitor combinations (ampicillin/sulbactam 2:1 ratio, aztreonam, carbenicillin, cefepime, cefoperazone, cefotaxime, ceftazidime, ceftriaxone, piperacillin, piperacillin/tazobactam constant four, ticarcillin, and ticarcillin/clavulanic acid constant two). Bacteria-antimicrobial suspensions were transferred immediately from the Sensititre® plates to flat-bottomed Nunc Edge 96-well plates, since the oCelloScope system (Philips BioCell, Allerød, Denmark) cannot measure round bottom plates. No discrepancies were associated with this step. All experiments on reference strains and clinical isolates were carried out in biological triplicates.

### Data analysis

The oCelloScope is a digital time-lapse microscopy technology that scans through a fluid sample generating series of images as previously described in 2013 by Fredborg et al. [13]. The oCelloScope instrument was placed inside an Innova 44 incubator (New Brunswick Scientific) allowing precise temperature regulation. As a result of the tilted imaging plane, the images recorded by the oCelloScope system constitute a parallelepipedum that forms the image stack. The projected z-stack image of a single z-plane was generated by combining the tilted images. Each well was scanned repeatedly every 15 min for 12 h. Time-lapse experiments, digital analysis, and image processing were conducted by a custom automation script in MATLAB (MATLAB Version: 8.0.0.783 (R2012b), The MathWorks Inc., Natick, MA, USA, 2000). Growth kinetics was determined by image stack processing based on contrast based Segmentation and Extraction of Surface Area (SESA). The Segmentation Extracted Average Length (SEAL) algorithm was designed to detect filamentation of rod shaped bacteria. SEAL determines the mean bacterial length and use contrast-based segmentation followed by morphological filtering on a projected z-stack image to measure the major axis length of each individual object. SEAL is limited when cells are overlapping and may lead to inaccurate determination of bacterial length at high cell concentrations or if bacterial filaments are crossing each other. Objects that spans the edge of the field of view are not included. GraphPad Prism version 6.00 for Windows, (GraphPad Software, San Diego, California, USA) was used for data analysis and graphing.

## Results and discussion

### Determination of bacterial length

**Fig. 2:**
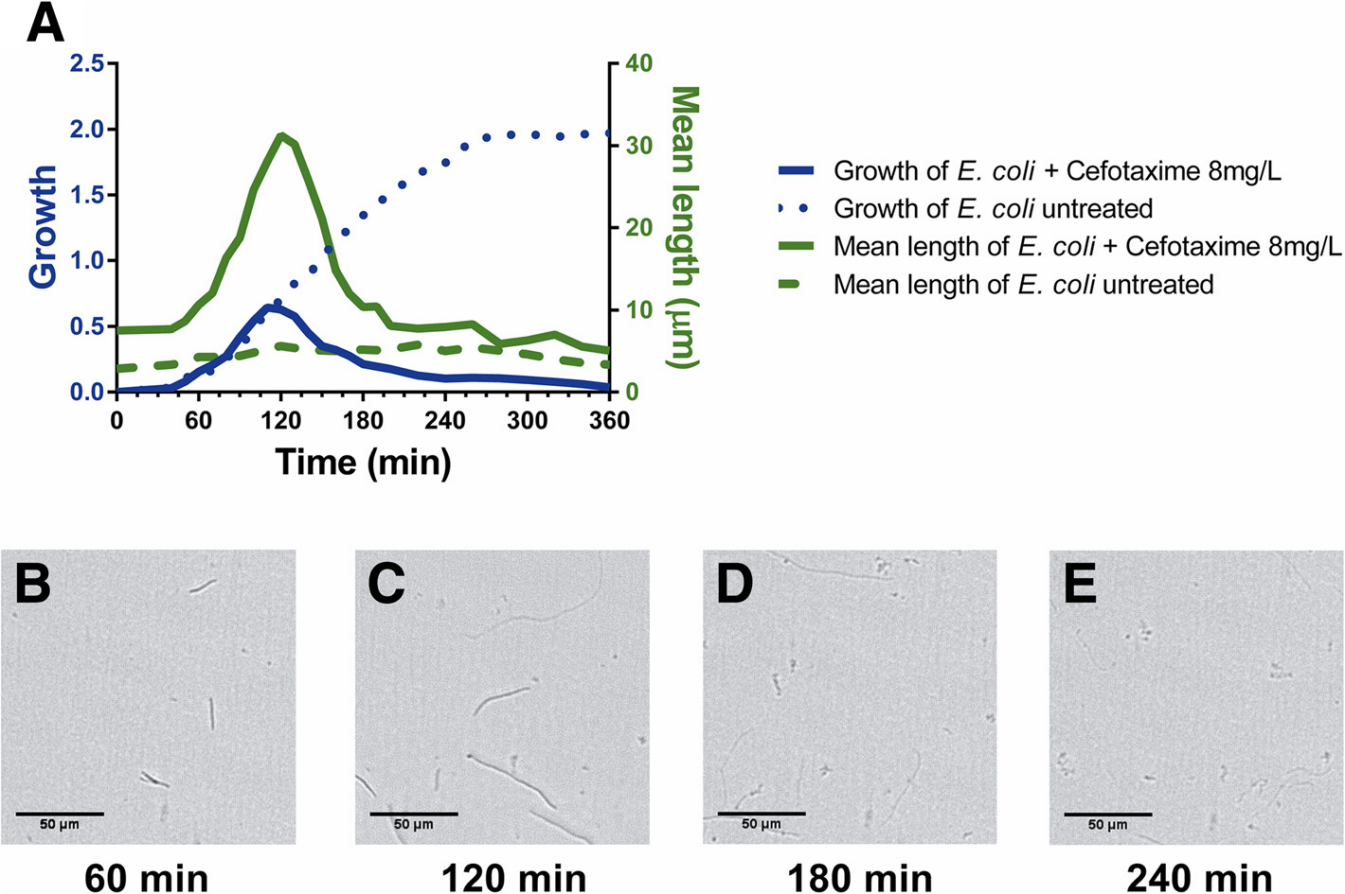
Effect of piperacillin on growth and length of *E. coli* obtained by the oCelloScope system. **a** Quantification of bacterial growth kinetics and filamentation was determined for untreated (*stippled lines*) and cefotaxime-treated (*solid lines*) *E. coli* by two different algorithms: Growth kinetics (*blue graphs*) was measured based on segmentation and extraction of surface area, and bacterial length (*green graphs*) based on segmentation extracted average length. Images of *E. coli* morphology treated with 8 mg/L of cefotaxime were obtained by the oCelloScope system at time **b** 60 min, **c** 120 min, **d** 180 min, and at **e** 240 min

Figure 1 shows antibiotic-induced filamentation in *E. coli* ATCC259222 by light microscopy. To examine the ability of the oCelloScope system to rapidly determine filamentation of *E. coli*, three *E. coli* strains were inoculated with a total of 12 β-lactam antibiotics or β-lactam–β-lactamase inhibitor combinations. Phenotypic antimicrobial susceptibility by the oCelloScope has previously been determined using the contrast based segmentation and extraction of surface area (SESA) algorithm [13]. However, when focusing on early detection of antimicrobial susceptibility within the first 60–120 min, antibiotic-induced bacterial filamentation may lead to deceptive interpretation of results. The *E. coli* reference strain was grown with and without the β-lactam antibiotic, cefotaxime. As seen in Fig. 2, filamentation affects growth kinetic measurements, because the algorithm is unable to distinguish between normal growth and filamentation, since both growth patterns resulted in an increased image area covered by bacteria – the value quantified by the algorithm. To be able to distinguish between these two growth patterns is particularly important when focusing on early determination of antimicrobial susceptibility, since this is complicated by filamentous bacteria as their elongated cell length may falsely be interpreted as normal growth. Consequently, we developed an algorithm to determine mean bacterial length of rod shaped bacteria based on segmentation extraction of average length (SEAL). In addition to measurements of growth kinetics, the *E. coli* reference strain was analyzed with the novel algorithm to determine bacterial elongation (Fig. 2a and Additional file 1: Movie S1). Morphological changes induced in the *E. coli* reference strain by cefotaxime were clearly visualized by the oCelloScope system after 30, 90, and 150 min of inoculation (Fig. 2b–d) and rapidly detected by the length quantifying algorithm (Fig. 2a, green stippled and full lines). The obtained results showed that treatment with cefotaxime initially increased the length of the *E. coli* reference strain drastically by inducing filamentation followed by lysis of the bacterial cells. Antibiotic-induced changes in *E. coli* morphology from elongation of cells to the production of ghost cells are a well-known phenomenon [14, 15]. Filamentation peaked at approximately 90 min with an average cell length of approximately 30 μm, (Fig. 2a, c). In concordance, β-lactam-induced elongation of cefazolin treated *E. coli* examined with dielectrophoresis demonstrated peaks in bacterial cell length after 120 min of incubation [16]. Examination of filament formation in both antibiotic resistant and sensitive *E. coli* showed that the filament formation was an initial response to cefotaxime occurring after 2 h of inoculation [17].

Even though 8 mg/L cefotaxime is the CLSI breakpoint concentration for ESBL-producing *E. coli* strains [18] and not for the *E. coli* reference control strain, it clearly demonstrates the point that if susceptibility of this strain was to be determined after 90 min based on the growth determining algorithm or systems relying on colorimetry or turbidometry, it would lead to the classification of this strain to be resistant to cefotaxime at the concentration of 8 mg/L. The ability of the length quantifying algorithm to differ between bacterial growth and filamentation, and in this situation clearly state that the latter is the case, enables correction of the falsely interpretation of filamentation as growth. Since filamentation is not directly correlated with antibiotic susceptibility or resistance, this cannot be determined at this time point, which is pivotal knowledge in relation to early AST.

### Early detection of β-lactam antibiotic-induced filamentation

A total of 12 β-lactam antibiotics or β-lactam–β-lactamase inhibitor combinations (ampicillin/sulbactam 2:1 ratio, aztreonam, carbenicillin, cefepime, cefoperazone, cefotaxime, ceftazidime, ceftriaxone, piperacillin, piperacillin/tazobactam constant four, ticarcillin, and ticarcillin/clavulanic acid constant two) were analyzed for their ability to induce filamentation in the three included *E. coli* strains (Table 1). These 12 β-lactam antibiotics included four (ceftazidime, aztreonam, cefotaxime, and ceftriaxone) of the five antibiotics recommended by CLSI to constitute the ESBL initial screening test [18].

**Table 1:** Effect of 12 β-lactam antibiotics or β-lactam–β-lactamase inhibitor combinations on antibiotic-induced filamentation (AIF) of three E. coli strains. The antibiotic concentrations of which filamentation is induced are given, if not detected for all concentrations (All) or if no filamentation is detected (N.D.; not detected). In addition, MIC values and the time required to detect them are included (time-to-result in minutes; TTR, (mean ± SE))

	*E. coli* ATCC 25922			ESBL-producing *E. coli*			Blood culture *E. coli*		
β-lactam antibiotics or β-lactam–β-lactamase inhibitor combinations	AIF	MIC	TTR	AIF	MIC	TTR	AIF	MIC	TTR
	conc.		(min)	conc.		(min)	conc.		(min)
ampicillin/sulbactam 2:1 ratio	2/1–8/4	4/2	97 ± 3.33	16/8	16/8	100 ± 0	2/1–8/4	≤2/1	113 ± 6.67
aztreonam	All	≤ 2	115 ± 5	N.D.	>16	65 ± 5	All	≤ 2	103 ± 1.67
carbenicillin	32–128	≤ 32	102 ± 1.67	N.D.	256	55 ± 5	All	≤ 32	60 ± 0
cefepime	2–4	≤ 2	103 ± 1.67	All	4	117 ± 3.33	2–4	≤ 2	132 ± 6.01
cefoperazone	All	≤ 4	117 ± 3.33	All	8	133 ± 6.67	All	≤ 4	155 ± 5
cefotaxime	4–8	≤ 4	102 ± 1.67	All	≤ 4	103 ± 1.67	4–8	≤ 4	93 ± 3.33
ceftazidime	All	≤ 1	133 ± 6.67	N.D.	>16	62 ± 1.67	All	≤ 1	117 ± 3.33
ceftriaxone	4–8	≤ 4	93 ± 3.33	All	≤4	115 ± 5	4–8	≤ 4	97 ± 3.33
piperacillin	All	≤ 8	103 ± 1.67	N.D.	>64	55 ± 5	All	≤ 8	163 ± 1.67
piperacillin/tazobactam constant four	All	≤ 8/4	113 ± 6.67	F	≤8/4	162 ± 1.67	All	≤ 8/4	115 ± 5
ticarcillin	All	≤ 8	123 ± 3.33	N.D.	>64	65 ± 5	All	≤ 8	100 ± 0
ticarcillin/clavulanic acid constant two	16/2–32/2	8/2	138 ± 1.67	N.D.	128/2	163 ± 1.67	16/2–64/2	≤ 16/2	102 ± 1.67

**Fig. 3:**
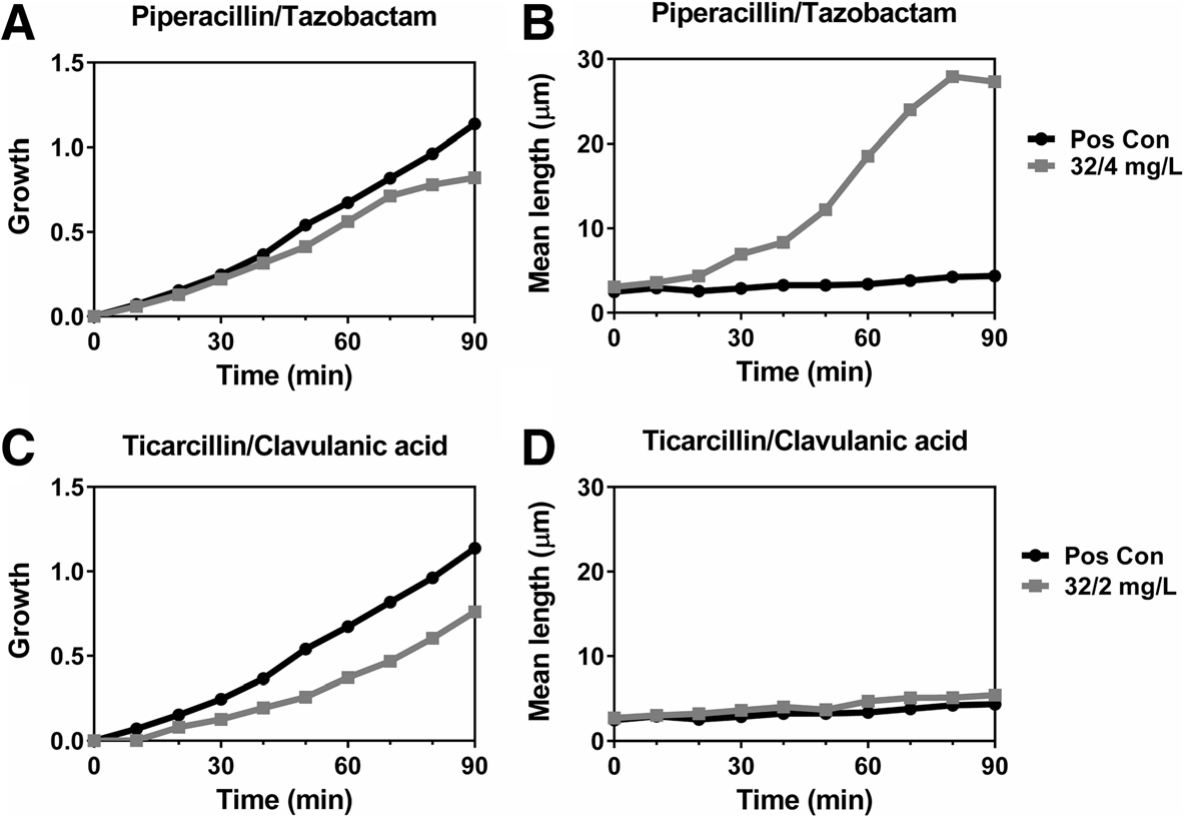
Early detection of antibiotic-induced filamentation of ESBL-producing *E. coli* by β-lactam–β-lactamase inhibitor combinations*.* Growth kinetics and mean bacterial length of *E. coli* treated with piperacillin/tazobactam (32/4 mg/L) (**a**–**b**) or ticarcillin/clavulanic acid (32/2 mg/L) (**c**–**d**). The piperacillin/tazobactam-induced filamentation (increased length >5 μm) can be determined within 30 min, while the absence of ticarcillin/clavulanic acid-induced filamentation can be determined within 40 min

The screening demonstrated significant variations in antibiotic-induced filamentation depending both of the specific antibiotic and the antibiotic concentration (from sub-minimum inhibitory concentration (MIC) and up to 5 × MIC). The use of the two algorithms (SEAL and SESA) in combination facilitates early detection of antibiotic-induced filamentation. Piperacillin/tazobactam-induced filamentation (increased length >5 μm) can be determined within 30 min, while the absence of ticarcillin/clavulanic acid-induced filamentation can be determined within 40 min (Fig. 3). Based on the absence of filamentation and the presence of normal growth when testing β-lactam–β-lactamase inhibitor combinations, the oCelloScope system holds the potential of rapidly determining the phenotype of β-lactamase producing bacteria – e.g. ESBL-producers by combining cephalosporins with β-lactamase inhibitors. Early determination of antimicrobial susceptibility is complicated in the case of filamentous bacteria such as *E. coli* as their elongated cell length may falsely be interpreted as growth in systems based on colorimetry or turbidometry (such as Vitek-2, Phoenix, MicroScan WalkAway). Consequently, the determination of β-lactam-mediated morphological alterations facilitates early appropriate targeted antibiotic therapy. The emerging interest in detection of β-lactam antibiotic induced morphological effects is seen by the recent development of techniques aimed at rapid detection of this phenomenon [16, 19–21].

**Fig. 4:**
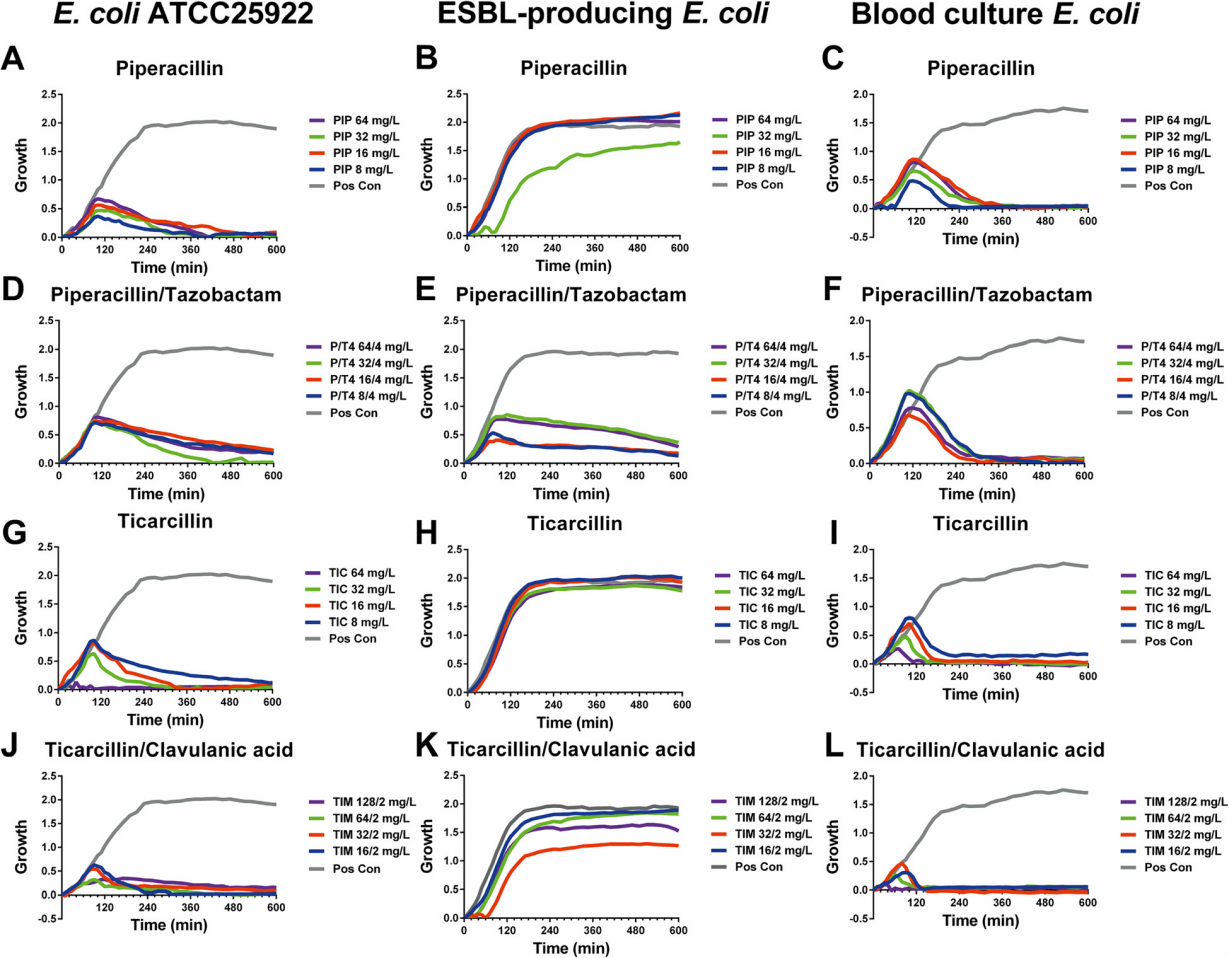
Effects of β-lactam–β-lactamase inhibitor combinations on *E. coli* growth kinetic. Reference strain *E. coli*, ESBL-producing *E. coli*, and a blood culture *E. coli* was treated with 4 different concentrations of either piperacillin alone (**a**–**c**) or with supplementation of tazobactam (**d**–**f**) as well as ticarcillin alone (**g**–**i**) or with supplementation of clavulanic acid (**j**–**l**). Growth kinetics were measured repeatedly every 15 min for 10 h

**Fig. 5:**
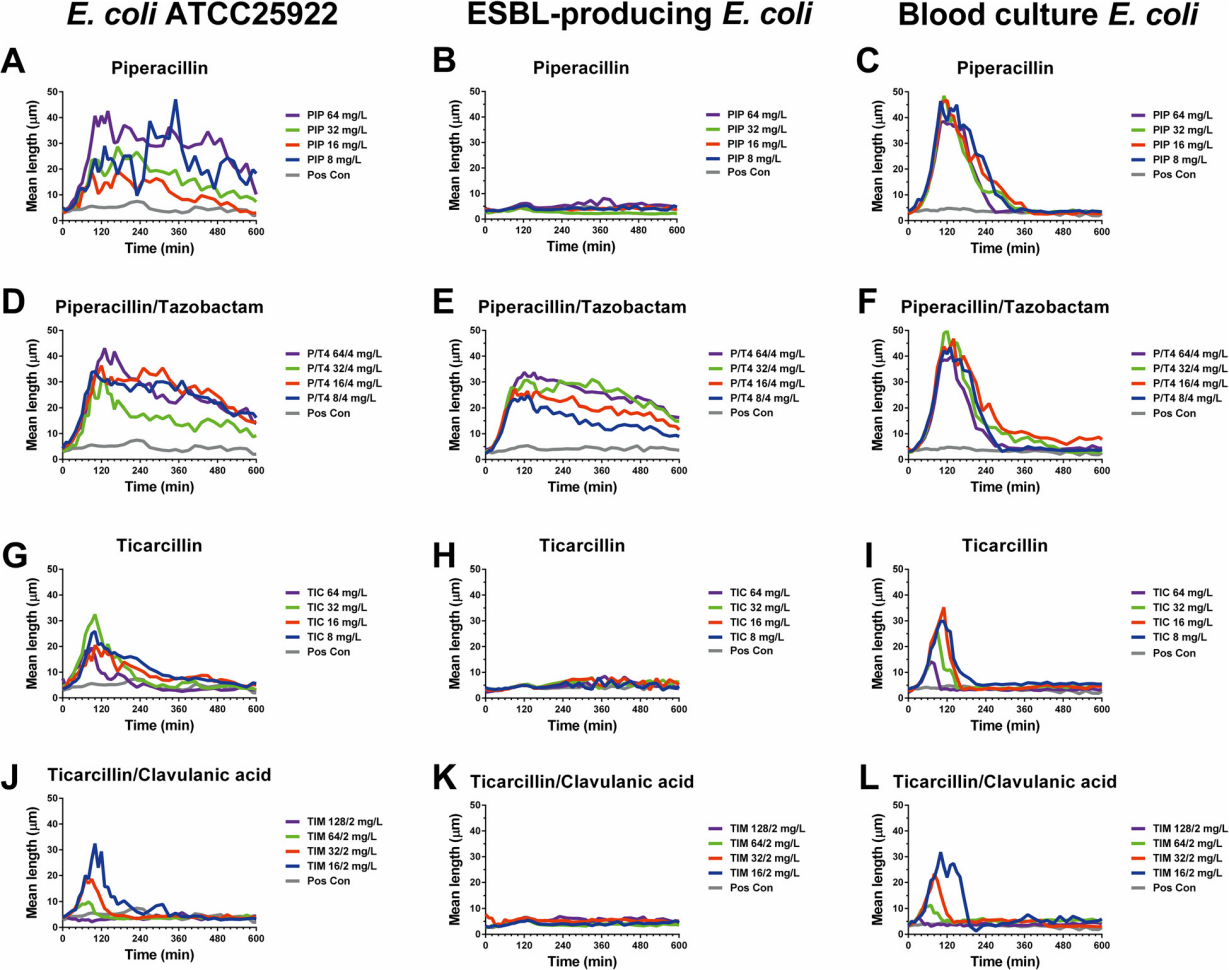
Effects of β-lactam–β-lactamase inhibitor combinations on bacterial length of *E. coli*. Reference strain *E. coli*, ESBL-producing *E. coli*, and a blood culture *E. coli* was treated with 4 different concentrations of either piperacillin alone (**a**–**c**) or with supplementation of tazobactam (**d**–**f**) as well as ticarcillin alone (**g**–**i**) or with supplementation of clavulanic acid (**j**–**l**). Bacterial length was measured repeatedly every 15 min for 10 h

The target for β-lactam antibiotics is cell wall synthesizing enzymes that have sensitivity towards penicillin, named penicillin-binding proteins (PBPs). The intrinsic β-lactamase resistance associated with PBP is an important mechanism in clinical isolates [22]. In *E. coli*, piperacillin exhibits high selective affinity towards PBP-3 and PBP2, as well as moderate binding to PBP1b [23–25]. Each PBP serves an essential role in bacterial cell life: PBP1a and 1b are responsible for cell elongation, PBP2 for cell shape, and PBP3 for cell division [25]. Inhibition of the latter results in cell filamentation, which is in concordance with the oCelloScope system detecting increased bacterial cell length for the *E. coli* reference strain and the blood culture *E. coli*, but not for the ESBL-producing *E. coli* when inoculated with 8–64 mg/L of piperacillin (Figs. 4 and 5a–c). Likewise, bacterial cell filamentation was detected by the oCelloScope system for the *E. coli* reference strain and blood culture isolate when inoculated with ticarcillin (8–64 mg/L), but not for the ESBL-producing *E. coli* (Figs. 4 and 5g–i). Ticarcillin preferentially binds to PBP-3 thereby inhibiting the last step of peptidoglycan synthesis in the septum formation leading to filamentation [8]. When analyzing the β-lactam–β-lactamase inhibitor combination, ticarcillin/clavulanic acid, increased bacterial cell length was observed for the *E. coli* reference strain and blood culture isolate in a dose–response manner in which the longest cell length was observed for the lowest concentration of the β-lactam antibiotic (Figs. 4 and 5j–l). No filamentation was observed for the ESBL-producing *E. coli*. Hence, this finding together with the growth detected by the SESA algorithm demonstrated that the combination therapy had no effect on the ESBL-producing *E. coli* (Figs. 4–5h and k).

## Conclusion

We presented an automated image-based algorithm which enables determination of antibiotic-induced filamentation. The method facilitates high-throughput screening of bacterial filamentation and thus has the potential to enhance antimicrobial susceptibility testing by providing this important knowledge to eliminate false susceptibility results in automated systems. The ability to determine the absence of filamentation and thereby the growth and resistance to an antibiotic enables the oCelloScope to be used as an early predictor of resistant bacteria. As for fast and accurate elucidation of filamentation and antibiotic resistance of clinical samples, it is recommended that the bacteria are in their exponential growth phase prior to addition of antimicrobials, and that the same growth media is used in the preparation and screening of the bacteria. Optimization of growth media to different bacterial strains and to the fastest rate of filamentation is endorsed.

## Additional file

Additional file 1:
**Piperacillin-induced filamentation in** ***E. coli*** **.** The time-lapse movies show *E. coli* treated with piperacillin (PIP, 64 mg/L) and without (Positive control), respectively; as well as graphs showing growth and average length of the bacteria. (MOV 4671 kb)

## Abbreviations

AIF: Antibiotic-induced filamentation
AST: Antimicrobial susceptibility testing
CAMHBT: Cation adjusted mueller-hinton broth with TES
ESBL: Extended spectrum β-lactamases
MIC: Minimum inhibitory concentration
N.D.: Not detected
PBPs: Penicillin-binding proteins
SEAL: Segmentation extracted average length
SESA: Segmentation and extraction of surface area
TTR: Time-to-result
